# Heritability and Artificial Selection on Ambulatory Dispersal Distance in *Tetranychus urticae*: Effects of Density and Maternal Effects

**DOI:** 10.1371/journal.pone.0026927

**Published:** 2011-10-31

**Authors:** Ellyn Valery Bitume, Dries Bonte, Sara Magalhães, Gilles San Martin, Stefan Van Dongen, Fabien Bach, Justin Michael Anderson, Isabelle Olivieri, Caroline Marie Nieberding

**Affiliations:** 1 Metapopulation, Conservation, and Co-evolution, Université Montpellier 2, Montpellier, France; 2 Evolutionary Ecology and Genetics, Biodiversity Research Center, Earth and Life Institute, Académie Louvain, Louvain-la-Neuve, Belgium; 3 Terrestrial Ecology Unit, Ghent University, Ghent, Belgium; 4 Centre for Environmental Biology, University of Lisbon, Lisbon, Portugal; 5 StatUA Statistics Center, University of Antwerp, Antwerp, Belgium; Institut Pasteur, France

## Abstract

Dispersal distance is understudied although the evolution of dispersal distance affects the distribution of genetic diversity through space. Using the two-spotted spider mite, *Tetranychus urticae*, we tested the conditions under which dispersal distance could evolve. To this aim, we performed artificial selection based on dispersal distance by choosing 40 individuals (out of 150) that settled furthest from the home patch (high dispersal, HDIS) and 40 individuals that remained close to the home patch (low dispersal, LDIS) with three replicates per treatment. We did not observe a response to selection nor a difference between treatments in life-history traits (fecundity, survival, longevity, and sex-ratio) after ten generations of selection. However, we show that heritability for dispersal distance depends on density. Heritability for dispersal distance was low and non-significant when using the same density as the artificial selection experiments while heritability becomes significant at a lower density. Furthermore, we show that maternal effects may have influenced the dispersal behaviour of the mites. Our results suggest primarily that selection did not work because high density and maternal effects induced phenotypic plasticity for dispersal distance. Density and maternal effects may affect the evolution of dispersal distance and should be incorporated into future theoretical and empirical studies.

## Introduction

In the context of a rapidly changing environment, the study of dispersal and its consequences is becoming ever more important. In particular, in order to predict the success of a species under increased fragmentation and climate change resulting in local extinctions, we must understand not only why individuals choose to stay or go but also how they can achieve successful colonization [1]. While emigration rate is an important factor to consider, the distances individuals disperse also affect gene flow and play an important role in predictive ecology [1], [2].

In general, most individuals move short distances while some move much further [3]. The “dispersal kernel” quantifies population-level dispersal distances. This term refers to the probability that a single dispersing organism will travel a certain distance before it settles [4]. The shape of the dispersal kernel, and therefore variation in individual probabilities of moving at given distances, can affect metapopulation dynamics [5], range expansion of invasive species [6], and colonization success of new habitats [7]. Importantly, theoretical models have shown that the individuals at the end of the tail can have different dispersal-related genotypes compared to individuals close to the origin of the distribution, therefore contributing to spatial heterogeneity of dispersal strategies [8], [9], [10]. In this regard, Haag et al (2005) [11] demonstrated that butterflies with a specific allele of the metabolic enzyme *phosphoglucose isomerase* (pgi) had higher flight metabolic rate. These individuals were shown to be in higher frequency in newly established populations and the authors suggest that these individuals have increased dispersal rate [11]. Therefore, we expect to see a genetic basis for differentiated phenotypes in dispersal and other behavioral traits [12].

Dispersal is considered to be the combined result of three distinct phases: emigration, inter-patch movement, and immigration [13], [14]. The motivations behind emigration have been well established, both theoretically and empirically. Organisms disperse because dispersal allows: (1) escape from competition by taking advantage of the temporal variability of their habitat [15], [16]; (2) escape from kin competition [17], [18], [19]; and (3) avoidance of inbreeding [20], [21]. The benefits to disperse are met with costs either directly or indirectly related to inter-patch movement [22] and immigration success [23]. Conditional dispersal strategies differ from ultimate causes of dispersal in that they respond to changes in the environment over the short-term and therefore take into account immediate changes in the cost/benefit ratio [24], [25]. Examples of these strategies, such as responding to population density [26] and different levels of kin-relatedness [27], may also influence departure from a patch.

In contrast to the wide variety of literature available on the evolution of emigration rate, only recently have theoretical studies focused on the evolution of dispersal distances [1], [4],[28],[29],[30],[31]. These studies generally propose that the factors affecting the evolution of emigration rate also affect the evolution of dispersal distance. For example, kin competition has been shown to favor long distance dispersal even with a very high cost to dispersal [29]. A distance-dependent cost of dispersal can still favor long distance dispersal as long as there is high habitat availability [4]. Importantly, because where an individual chooses to settle can have direct fitness consequences, theory suggests that dispersal distances are subject to natural selection [31] and can therefore evolve [30]. However, to our knowledge, no theoretical or empirical studies have been done that examine the effects of conditional dispersal strategies, such as density dependence, on dispersal distance or the dispersal kernel.

To start filling this gap, in the present study we selected on dispersal distance using *Tetranychus urticae*, a generalist herbivorous mite of high economical importance [32], [33]. Understanding the dispersal behavior of this pest species in greenhouses is fundamental to improving biological control techniques because the effectiveness of predators depends on the spatial distribution and density of the prey [34], [35]. *T. urticae* disperses individually by walking from one plant to another [36], [37], or aerially by positioning their bodies in such a way as to catch wind [38]. Under extreme conditions (overcrowding coinciding with food depletion), individuals gather at the plant apex to form a ball made by mites and silk threads [39]. Newly emerged mated females are the stage most likely to disperse individually, through either aerial or ambulatory means [40], [41]. Aerial dispersal in this species has been shown to be heritable, respond to selection on increased and decreased demonstration of the behavior, and be negatively correlated with fecundity, although not consistently [42]. Ambulatory dispersal propensity has been shown to respond to artificial selection and shows a trade-off between dispersal and life-history traits (diapause and fecundity) in one study [43] but not in another [44]. However, these three studies focused on emigration (or dispersal propensity) and did not include dispersal distances.

Artificial selection on ambulatory dispersal distance (rather than dispersal propensity) has arguably never been performed (Table 1). Furthermore, in the present study we took into account several additional considerations to match the current theoretical framework on dispersal. This includes the distinction between the general condition of individuals (*sensu* David *et al.*, 2000) and their ability to disperse. Indeed, most artificial selection studies on dispersal select individuals that leave or stay on a patch, preventing the researchers from determining if the individuals that remain did so by choice or due to a general condition (eg sickness) that impedes their movement. Unlike other studies which produced low dispersing individuals by selecting those individuals that do not move from their original patch, we selected only among individuals that left their natal patch (similar to the field studies of Haag *et al.*, 2005 and Niitepold *et al.*, 2009). After ten generations of selection in nine independent replicates, we assessed variation in dispersal distance and its correlation with fecundity, longevity, sex-ratio, and developmental time in high dispersing (HDIS), low dispersing (LDIS), and Control (C) treatments. We finally tested if there was heritability for dispersal distance at both a high and low density. These experiments allowed us to test (1) if dispersal distance responds to artificial selection, (2) if selection on dispersal was linked to a correlated response of other life-history traits, and (3) whether dispersal distance is affected by density.

**Table 1:** Review of articles that report a response to artificial selection based on a dispersal trait with accompanying heritability values, when available.

Organism	Dispersal trait selected	*h^2^*	Reference
*Gryllus firmus* (Sand cricket)	flight propensity	wing dimorphism 0.65 (Roff 1986b).	Fairbairn & Roff (1990)
*Tribolium confusum* and *Tribolium castaneum* (flour beetle)	emigration		Ogden (1970)
*Tribolium castaneum* (beetle)	flight propensity		Diez & Lopez-Fanjul (1978)
*Tribolium confusum* (flour beetle)	emigration		Korona (1991)
*Tribolium confusum* (flour beetle)	emigration		Lomnicki (2006)
*Epiphyas postvittana* (moth)	flight duration	0.56 parent-offspring regression; 0.53 using breeders equation	Gu & Danthanarayana (1992)
*Cydia pomonella* (codling moth)	mobility	0.29 for females and 0.43 for males	Keil *et al.* (2001)
*Tetranychus urticae* (2 spotted spider mite)	emigration	0.28	Li & Margolies (1994)
*Tetranychus urticae* (2 spotted spider mite)	emigration		Yano & Takafuji (2002)

## Materials and Methods

### Laboratory population

The base population was composed of the “LS-VL” strain of *T. urticae* spider mites [45]. The LS-VL strain was originally collected in October 2000 from roses in a garden near Ghent, Belgium and since then maintained on potted *Phaseolus vulgaris* plants variety ‘Prelude’, named “bean” hereafter, in a climatically controlled room at 26.5±1C, 60% RH and 16/8 h (L/D) photoperiod with a population size of about 5000 mites [46]. In November 2008, the strain was transferred to Montpellier, France, starting from approximately 800 mites and maintained under the same conditions with a population size of approximately 2,500 mites. Bean seeds (from Vlaamszaadhuis Belgium) were sowed once per week and cultured in an herbivore-free greenhouse at 25°C.

### Artificial Selection for high and low dispersal distance

**Figure 1:**
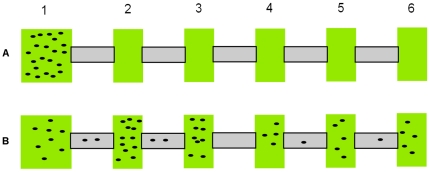
Artificial selection set-up. Schematic representing the artificial selection procedure. 150 mated young females were placed on patch one (a). The females dispersed through the linear system for 48 hours (b), at which time 40 females were removed from patches five and six for the HDIS treatment, from patches two and three for the LDIS treatment, and randomly from all patches for the Control treatment. Mites are represented by black circles.

Fifty females from the LS-VS strain were collected and allowed to lay eggs for 48 hours on a fresh bean leaf (7 cm×7 cm). When their synchronized offspring hatched, 150 one to two-day old mated females were chosen at random to start the selection procedure. No males were used in the selection experiments because females are considered to be the dominant dispersers in this species [40], [47]. The first generation of selection was performed by placing the females on a starting fresh bean leaf (2 cm×2 cm) and allowing them to settle for 30 minutes. This leaf was then connected linearly to five consecutive bean leaves (each 2 cm×1 cm) via Parafilm bridges (8 cm×2 cm), forming a “six-patch” line system (Fig. 1). This distance was chosen because pilot experiments revealed that on average no more than 20% of the mites reached the sixth patch after 24 hours. The mites were allowed to disperse from patch one to the five other patches for 48 hours. Therefore, each mite that disperses to the next patch must make a settlement decision and choose to advance to the next patch, remain at the current patch, or return to the previous patch. At the end of this first trial, a total of 40 females were selected from patches five and six and placed on a new fresh bean leaf to create the first generation of the “high dispersal” treatment (HDIS). Similarly, a total of 40 females were selected from patches two and three and placed on a new bean leaf to create the “low dispersal” treatment (LDIS). A “Control treatment” was produced by randomly picking 40 females among the six-patch line system according to the proportion of females found on each patch. The 40 HDIS, LDIS and Control females were allowed to lay eggs for two days and their synchronized offspring were used to produce the second generation of selection. 150 one to two-day old mated female offspring from each HDIS, LDIS and Control treatment were placed at the start of three independent “six-patch” line systems respectively, named hereafter HDIS, LDIS, and Control treatment systems. The mites were allowed to disperse for 48 hours, after which 40 females were selected from patches five and six of the HDIS treatment system and the procedure described above was repeated to produce the third generation of HDIS treatment. Similarly, for the LDIS and Control treatments, 40 females were selected from patches two and three of the LDIS treatment system, or randomly picked from the Control treatment system, respectively. In total, three replicates of each HDIS, LDIS and Control treatment were produced from the base population. All nine replicate lines were started within two weeks of each other, and throughout the selection procedure the trials naturally desynchronized depending on the developmental time of the mites.

Response to selection over ten generations in the three selection treatments (HDIS, LDIS and Control) was tested by modeling differences in dispersal, with generation time as a continuous factor to test the strength and direction of selection in each line. Dispersal distance was modeled either as continuous trait, so modeling the average covered distance (average leaf disc number) or as a multinomial trait, so testing whether the proportion of mites moving to one of the five patches differed between treatments. The factor replicate was nested within treatment and the interaction replicate*generation were considered random factors. Responses on average covered distance were modeled by mixed models with a Gaussian error structure (proc mixed; SAS Institute 2003): multinomial models were fitted using clogit-link (proc Glimmix; SAS Institute 2003). Effective degrees of freedom were approximated by the Satterthwaite procedure.

### Estimating correlations of dispersal distances between generations

At the end of the selection experiment, we performed an autocorrelation analysis based on the residuals from a mixed-model (using the nmle package in the open source software R 3.1-97). We performed this analysis after observing a pattern in the distribution of mites in each generation of selection. Our dependent variable was the proportion of mites found either on patches one and two or on patches five and six. For the explanatory variables, generation was a fixed continuous factor, treatment was a fixed categorical factor, and replicate was a random factor. We aimed to determine if the proportion of mites found either on patches one and two or on patches five and six in a given generation affected the proportion of mites found on the same patches in subsequent generations. Using the residuals from the mixed model, we performed an autocorrelation analysis [48].

### Correlated responses in life-history traits

Trade-offs in resource allocation between different fitness traits can be measured to assess whether evolutionary change after artificial selection has taken place [42], [43], [49], [50], [51]. To test this, the following traits were measured in females from all replicates and treatments after ten generations of selection: developmental time, fecundity, sex-ratio, and longevity. Forty mated females were kept on bean leaves and life-history traits were measured in their offspring. For each of the nine replicated treatments, a total of 70 eggs were placed individually on bean leaves (1.5 cm×1 cm) and allowed to fully develop. Developmental time was recorded as the period (number of days) between egg hatching and first oviposition. Females in their last molt were mated to a male from the same replicate and allowed to lay eggs for a total of six days. Every two days each female was given a new leaf and eggs were counted to measure fecundity. Offspring from the first four days of oviposition were allowed to fully develop to obtain sex-ratio data. Longevity was estimated by recording the date of female death. Differences in life-history traits among selection treatments were tested with a linear mixed-effect model procedure (GLMM using the lme4 package by Douglas Bates in the open source software R 2.5.0) with selection treatment as a fixed factor and replicate nested within selection treatment as a random factor. The proportion of females to males was analyzed using a mixed logistic regression with a binomial error structure. For the analysis of sex ratio data, only cases in which females laid eggs each day for the first four days were used for analysis. The longevity analysis was performed using the ProcGlm in SAS with block nested within treatment.

### Heritability of dispersal distance at high and low density

To estimate heritability of dispersal distance and the effect of density on heritability, we performed parent-offspring regressions [52] at high (150 individuals) and low (ten individuals) density. The high density experiment mimicked the artificial selection experiments. Using the base population, we placed 150 mated females of synchronized age (one to two-days old) and allowed them to disperse over six patches for 48 hours. After this, 82 mothers were placed on bean leaves and left to oviposit for 48 h. Simultaneously, 250 females were collected from the base population and left to oviposit for the same time period. Each tested mother that had at least five to seven female offspring within one day old of each other were used in the experiments. These female offspring were also mated with males from the base population. They were then marked with a water color (Royal Talens, Apeldoorn, Holland), which does not affect the behavior of mites [53], [54], and divided among different leaves (4 cm^2^). We placed a maximum of two sisters on one leaf and a maximum of eight families were represented on each starting bean leaf. Subsequently, the offspring of mites from the base population of the same cohort as the offspring of tested mothers were also placed on the starting leaf to create the same density as found in the first part of the heritability experiment and the artificial selection experiments. After 30 minutes, the starting leaf was attached linearly to five other bean leaves (2 cm×1 cm) using Parafilm bridges (8 cm×2 cm) to form a six-patch line system.

In the low density heritability experiment, 100 one to two-day old females from the base population were mated randomly with males. The dispersal propensity of 100 females was measured by placing ten groups of ten females each on a starting bean leaf (1 cm×1 cm) and allowing them to settle for 30 minutes. The starting leaves were then connected linearly to three consecutive bean leaves (1 cm×1 cm) via Parafilm bridges (8 cm×1 cm). Females were allowed to disperse among the leaves for 48 hours, after which the position of each female on the four different leaves was recorded. Each female was then reared individually and allowed to lay eggs for 48 hours. If females produced at least ten female synchronized (emerging within 48 hours of each other) offspring, these offspring were also mated with males from the base population and one group of these ten female offspring was used to test dispersal propensity as described above.

For both heritability experiments, the mean value of the offspring was used to perform a parent-offspring regression (Falconer and Mackay, 1996, pp 160–166). The slope of the regression equals half of the heritability estimation as the trait was measured in only one parent. A linear model was used to calculate the heritability and standard error (SE) in the open source software R 2.5.0.

## Results

### Characterization of the LS-VS strain

The LS-VL strain was chosen because microsatellite analysis data revealed that the population was reasonably polymorphic: observed heterozygosity was 0.45±0.28 (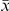 ± SE) in a sample of ten (diploid) females at ten microsatellite loci, with two to seven alleles per locus (I. Olivieri et al., unpublished data). Also, LS-VL strain is known for its ability to become quickly resistant to acaricides and fungicides [55]. These two observations led us to hypothesize that this population would have enough genetic variation to respond to artificial selection on dispersal distance.

### Artificial selection for increased and decreased dispersal distance

**Figure 2:**
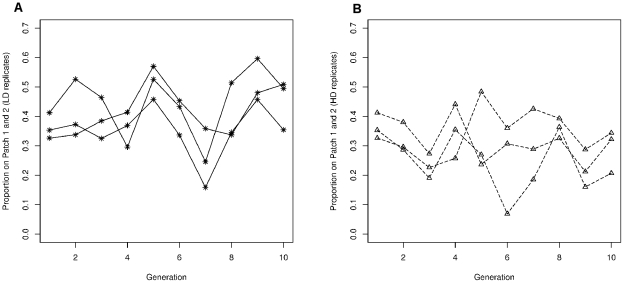
Effect of artificial selection on the proportion of females found on patch one and two. Proportion of females found on patch one and two after 48 hours by generation and by treatment: (a) LDIS (b) HDIS.

No interaction between the three selection treatments and generation was observed, indicating no significant changes in the mean dispersal distance or the proportion of individuals settled on each patch over the ten generations (Figure 2a,b, table 2). Variation among lines was rather small (see variance components; table 2).

**Table 2:** Results of mixed models for dispersal distance over ten successive generations (fixed effects and variance components).

*Gaussian model*							
	num df	den df	F	P-value	varcomp	mean	se
Treatment	2	6.22	1.23	0.35	σ^2^_line_	0.052	0.041
Generation	1	6.1	0.33	0.58	σ^2^_lineXgeneration_	0.002	0.001
Treatment × Generation	2	6.1	0.44	0.62	σ^2^_residual_	3.061	

*Multinomial model*							
	num df	den df	F	P-value	varcomp	mean	se
Treatment	2	6.20	0.33	0.58	σ^2^_line_	0.045	0.037
Generation	1	6.36	1.39	0.31	σ^2^_lineXgeneration_	0.002	0.001
Treatment × Generation	2	6.20	0.35	0.71			

Num df and den df represent the numerator and denominator degrees of freedom. Mean is the mean variance explained by the random effect, and se represents the standard error of the variance.

### Correlation between generations

**Figure 3:**
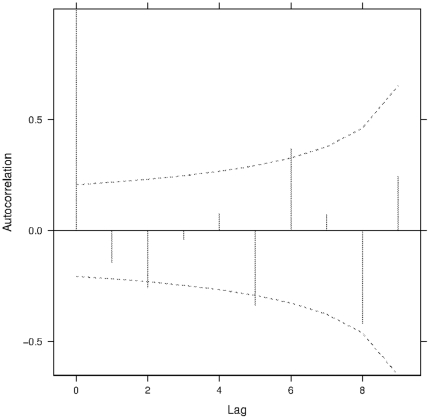
Correlation between generations. Autocorrelation between generations of the proportion of individuals found on patches one and two for all pooled replicates and treatments. Dotted lines represent the confidence intervals at 0.95. Lag time indicates the generation time passed between comparisons.

We observed a significant negative correlation between every second and fifth generation when looking at the proportions of individuals found on patches one and two. In other words, the proportion of individuals on the first two patches was negatively correlated with the proportion of individuals found on those two patches every two generations and five generations later (Fig. 3). We saw no significant correlations between any generations when looking at the proportion of mites found on patches five and six.

### Correlated responses in life-history traits

The average developmental time in days among replicates was 6.83±0.09 (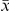
± se) for the HDIS treatment, 6.94±0.12 days for the LDIS treatment, and 6.73±0.07 days for the Control treatment. Developmental time was not significantly different among selection regimes (likelihood ratio test = 0.0186, d.f. = 2, p = 0.99). Therefore, the selection regime experienced for ten generations did not affect their developmental time.

The average fecundity among replicates was 59.15±1.18 eggs for HDIS, 56.79±1.14 eggs for LDIS, and 58.79±1.23 eggs for Controls. Fecundity was not significantly different among selection regimes (likelihood ratio test = 0.1536, d.f. = 2, p = 0.93).

The proportion of females to males was not significantly different among selection regimes (likelihood ratio test = 2.867, d.f. = 2, p = 0.24). The average proportion of females to males was 0.74±0.074 for HDIS, 0.71±0.064 for LDIS, and 0.71±0.065 for Controls.

Among females, longevity was not significantly affected by the selection regime. Least-square mean longevity in days of females was 22.65 days for HDIS, 23.25 days for LDIS, and 24.97 days for Controls. Males had an overall significantly longer life-span regardless of selection regime: least-square mean longevity is 23.42 for females and 28.9 days for males (*F_2,4_* = 18.99, p<0.001) regardless of selection regime.

### Estimation of heritability of dispersal distance at high and low density

**Figure 4:**
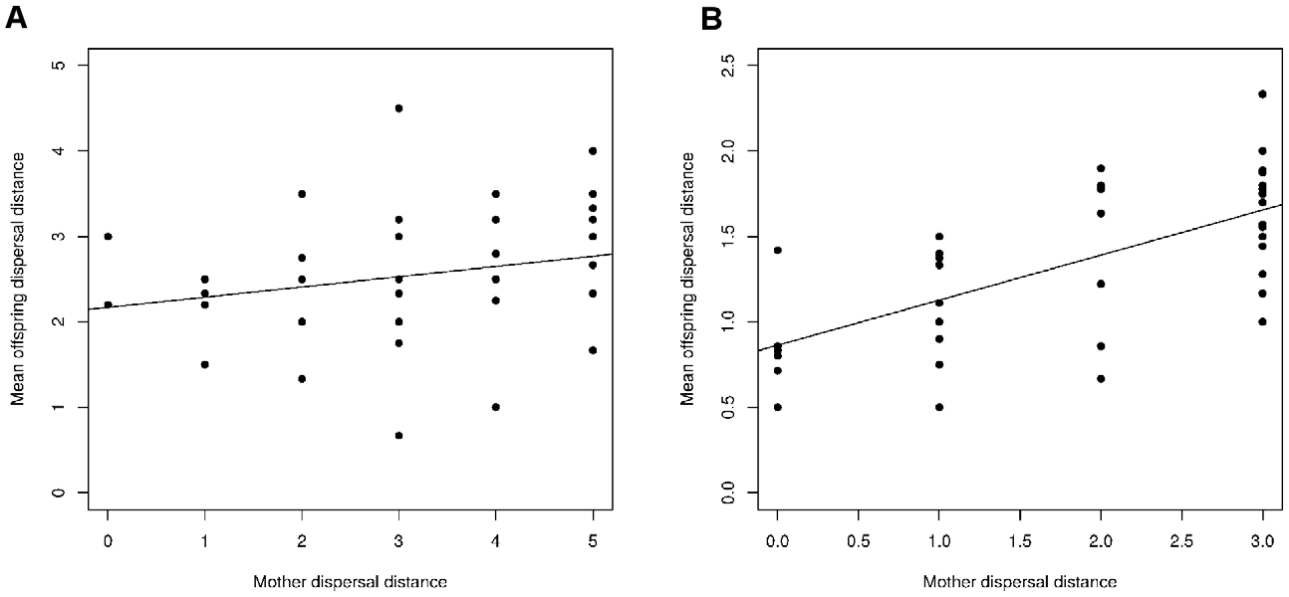
Mother-daughter regression of dispersal distance. (a) High density (150 mites) and (b) low density (10 mites).

To test the effects of density on the heritability of dispersal distance, we performed two parent-offspring regressions at both a high and low density. Of the initial 82 females tested from the base population for the high density experiment, 50 yielded offspring with at least eight females of synchronized age which could be used for the estimation of heritability of dispersal distance. A parent-offspring regression revealed a non-significant relationship between the distance travelled by the mother and the mean distance traveled by her offspring (n = 50, *h* ^2^ = 0.24±0.16, p = 0.18, Fig. 4a).

Of the initial 100 females tested from the base population for the low density experiment, 43 yielded offspring with at least ten females of synchronized age. Mothers that moved to further patches produced daughters that also moved further (Fig. 4b) and there was significant additive genetic variability for the trait, as indicated by the high and significant heritability estimate for the mean dispersal distance (*h* ^2^ = 0.52±0.12, p<0.001).

## Discussion

Under controlled conditions, we performed artificial selection for dispersal distance during ten generations in *T. urticae*. To determine whether trade-offs were present between dispersal distance and other traits associated with fitness, we also performed an extensive measurement of life-history traits, yet we did not observe such trade-offs. Despite indication of genetic variability for dispersal in *T. urticae* [43] and other organisms (Table 1), we did not detect a response to selection in terms of mean dispersal distance nor proportions of mites found in successive patches in the two directions of selection. We did, however, observe that maternal effects could be involved in the between generation dispersal behavior in this species (Fig. 4). We also observed that heritability for dispersal distance depended on the density at which the experiment took place. Our results thus suggest that the inclusion of external (environmental) factors (sensu Clobert 2009) and their potential interactions with dispersal distance prevented successful selection on dispersal in this study.

We avoided common methodological flaws that, to our knowledge, may have prevented efficient selection to take place in our setup. First, the base population was chosen because microsatellite data indicated the presence of genetic variability, suggesting that additive variation for dispersal and other life-history traits should be present. Second, the population size used to generate the next generation of selection was maintained at 40 in each replicated line. Previously, successful selection on emigration rate in *T. urticae* was performed using only 20 individuals per replicated line [43]. To increase selection pressure, small sample sizes for starting the next generation are commonly used but usually no less than 30 individuals per generation [42], [56], [57]. Our sample size may be sensitive to genetic drift and therefore it is possible that a high or low dispersing genotype will become fixed in a population. However, since we did not observe significant differences in dispersal distances or life-history traits between replicate lines within treatment, it is unlikely that genetic drift played a role in our experiment. Third, we also maintained three control lines by choosing individuals randomly from among the six patches. Fourth, we have selected on a behaviour that is commonly utilized by populations of *T. urticae* in cultures and greenhouses so that we selected on a natural behavior. Indeed, ambulatory dispersal is common in fields and in greenhouses for inter-plant movement in *T. urticae* [41], [58]. We additionally used one to two-day old mated females, which are recognized as the dominant dispersers in spider mites [38], [40], [43]. These experimental conditions aimed at maximizing the efficiency and quality of our artificial selection procedure.

Even so, selection was not successful and there are several explanations. It is possible that the selection gradient imposed in our experiments was not high enough to warrant a response. Yano (2002) was successful at performing artificial selection using only a two patch system; we attempted to use an elongated version of this system to further tease out long and short distance dispersers. In nature, mites are capable of walking at a speed of 6 m/hour [33]. Because the experiments were not monitored under constant surveillance, we also do not know to what extent the mites traveled back and forth between patches, and this is the same in the heritability experiments. However, our experiments were not testing metabolic activity or selecting on how far mites were capable of moving. Rather, we estimated the minimal number of dispersal events that mites had performed after 48 hours. Since there were six patches in the system, mites arriving on the sixth patch had to make at least five independent decisions of dispersal. Therefore, we selected on mites who chose to settle closer or further away from the starting patch. These dispersal conditions were similar between the heritability and selection protocols and thus cannot be responsible for the unsuccessful selection procedure.

We show that density played a major role in the results of our experiments. Indeed, at a high density, similar to our artificial selection experiments, no narrow-sense heritability on dispersal distance was observed, while at a lower density, heritability was significant. The same trend is observed, however in the high density heritability test the slope is lower and the variance is higher. Density differences could explain why a lower *h^2^* value was observed despite significant additive genetic variability for the trait. It has been shown that heritability may disappear because of an increase in environmental variance under stressful conditions [52], [59], [60]. Empirical studies show a trend for lower heritability in stressful, unfavorable conditions [61]. Although *T. urticae* has a higher fitness when living in small groups [62], it may well be that the density in our experiment exceeded the optimal density for *T. urticae*. Such high density could interrupt feeding behavior and increase competition for resources, thus creating a stressful environment.

Performing the selection experiments at a lower density would certainly be useful. Our results indicate high heritability and more variability for dispersal distance under lower densities. Furthermore, the selection gradient would be increased by selecting on fewer individuals. However, low density conditions can present other complications. For example, low population densities can increase the level of inbreeding, which could in turn confound heritability estimates by reducing genetic variation and increasing the chance of inbreeding depression [63]. These conditions also increase the chance of genetic drift, further minimizing variability for the trait [64], [65]. The complications make for a difficult scenario in any selection experiments that use too small sample sizes, and are a specific complication for artificial selection experiments that attempt to select on dispersal distance based on density.

In conjunction with density as an immediate driver of conditional strategies, maternal effects related to density can explain the retrieved negative autocorrelation between every second and fifth generation in our artificial selection experiments. This pattern is visible for both the HDIS lines and the LDIS lines when looking at the movements of the mites across generations (Fig. 3a,b). These results cannot be due to common environmental effects because the experiments were not temporally synchronized. Maternal effects may be an adaptive mechanism of phenotypic plasticity in which the mother can influence the phenotype of her offspring based on cues from her environment [66]. Maternal effects have been shown to affect offspring performance [67], diapause induction [68], and aerial dispersal behavior in this species (D. Bonte, unpublished data). Our results indicate that the density experienced by the mother could have influenced the dispersal distances of her offspring (Fig. 3a,b). Specifically, when the mother experienced high density during the experiments, her offspring dispersed further to escape from the high population density. On the other hand, when the density experienced by the mother was low, her offspring dispersed less. In our experiments, the mothers experienced high density on patches one and two and this led to a significant decrease of individuals found on those patches two generations later. The reverse was true as well. The same pattern on patches five and six was not observed, likely because the quality of the last patches was always higher than the first two patches. Furthermore all individuals in the system must pass through patch one and two while not all individuals are obliged to pass to patches five and six. We are prudent with our results because we have only ten generations and thus few data points with which to do the analysis. Even so, the analysis shows a cyclical trend indicating a negative correlation between every two generations.

Correspondingly, maternal effects could explain the presence of significant heritability in the mother-daughter regressions (Fig. 2a,b). In further tests on heritability in *T. urticae*, it might be more useful to perform sib-analysis in order to exclude the influence of maternal effects [52], [69]. Yet, the high density heritability test did not reveal the presence of maternal effects. This is most probably because, as suggested by the results of our artificial selection experiment, maternal effects were seen only after two generations.

In addition to density and maternal effects, the genetic relatedness of individuals and therefore kin competition could have influenced the dispersal behavior of the mites [17], [19] in the artificial selection experiments. While all individuals were genetically unrelated at the start of the selection experiments, successive generations of selection might have modified the genetic composition of the lines. While highly inbred individuals will avoid each other [70], close relatives are attracted to each other through the silk (Clotuche et al, unpublished data). Kin competition could therefore vary through time and might have affected the outcome of the selection procedure on dispersal in our setup. Increasing kinship in the lines might have either decreased dispersal propensity in the case that the mites are attracted to the silk of kin [71], or it might have increased dispersal propensity in case inbreeding avoidance strategies are present in the species [27], [70], [72].

The results of our study can have major implications for future theoretical studies [73], specifically those attempting to model the evolution of dispersal distances during range expansion. Travis et al (2009) investigated the evolution of density-dependent emigration strategies at an expanding range margin and showed that moderate dispersal rates are expected to evolve even at low densities [74]. Burton et al (2010) showed that at the range margin, dispersal and reproduction are selected for at the cost of competitive abilities [75]. Both of these models could be readily expanded to include the evolution of reaction norms in dispersal distance along with maternal effects. These reaction norms would be based on the idea that heritability for dispersal distance decreases as population density increases, and that at low densities the genetic component of dispersal plays a larger role than the environment [76]. Furthermore, in the first theoretical attempt to model dispersal distance as a function of population density in an actively dispersing species, Poethke et al (2011) showed that in species which use an informed dispersal strategy, increases in population density also lead to increases in dispersal distance [77]. This study, along with our results which indicate that at high density the environment plays a larger role in determining dispersal distance than the genetic component, could be expanded to predict the evolution of dispersal distance and invasion rates at the expanding range of a species.

In conclusion, we emphasize the rarity of empirical studies which focus on the evolution dispersal distance. We show that population density and density-dependent maternal effects are influential in the strength and direction of the evolution of this trait, and suggest that density can induce phenotypic plasticity for dispersal distance. A clear understanding of how heritability of dispersal is affected by population density will allow us to more accurately study metapopulation dynamics, colonization, and invasion processes. Furthermore, maternal effects and heritability of dispersal distance as a function of population density should be incorporated in theoretical and empirical studies on dispersal distance as well as dispersal kernels at range margins. We suggest that further studies incorporate our findings in order to provide a fuller picture of the evolution of dispersal distance.
